# A prospective observational study to assess the compliance to enhanced recovery protocols among patients undergoing bone and soft tissue cancer surgeries and their postoperative outcomes

**DOI:** 10.1007/s13193-025-02374-w

**Published:** 2025-07-22

**Authors:** Ashwini D. Rane, Sohan Lal Solanki, Sagar Apte, Priya Bhatia, Jeson Rajan Doctor, Prakash Nayak, Ashish Gulia, Ajay Puri

**Affiliations:** 1 Department of Anaesthesiology, Critical Care and Pain, Advanced Centre for Treatment Research and Education in Cancer, Tata Memorial Centre, Homi Bhabha National Institute, Navi-Mumbai, India; 2 Department of Anaesthesiology, Critical Care and Pain, Tata Memorial Hospital, Homi Bhabha National Institute, Mumbai, India; 3 Bone and Soft Tissue Services, Department of Surgical Oncology, Tata Memorial Hospital, Homi Bhabha National Institute, Mumbai, India; 4 Department of Surgical Oncology, Homi Bhabha Cancer Hospital and Research Centre, Tata Memorial Centre, Homi Bhabha National Institute, New Chandigarh, India

**Keywords:** Enhanced Recovery after Surgery, Bone and Soft Tissue, Cancer Surgeries, Compliance, Post-operative Outcomes

## Abstract

The Enhanced Recovery After Surgery (ERAS®) protocols are designed to maximize postoperative recovery and reduce complications. Despite extensive research on ERAS®, its implementation in bone and soft tissue surgeries is under investigated. The study aimed to assess the percentage compliance with individual components of the ERAS® pathway, determine the average overall compliance among patients, and compare postoperative complications across varying levels of compliance. In 300 patients with bone and soft tissue cancer surgeries from December 2021 to May 2023, we measured 20 ERAS® components for compliance including preoperative, perioperative, and postoperative items. We computed compliance percentages and postoperative results like postoperative length of stay, postoperative complications as per Clavien-Dindo (CD) class of complications, and readmission rates. The overall compliance rate with ERAS® protocols was 84.5% (81.8% in bone tumour groups and 82.2% in soft tissue groups). A significant correlation was observed between higher compliance and better outcomes. Patients with higher compliance had a shorter median hospitalstay of 7 days compared to 10 days for those with lower compliance (p < 0.001). Additionally, 70.3% of patients mobilized with limb physiotherapy within 24 h of surgery, and 98.7% received multimodal analgesia. Majority (81.7%) of the patients experienced minor complications (CD class I-II), and 5.6% experienced major complications (CD class III-V). Higher ERAS® compliance was associated with shorter hospital stays and fewer complications, suggesting its potential of better postoperative outcomes in bone and soft tissue cancer surgeries. Further studies stratifying patients by surgical complexity are needed to confirm these findings. Clinical Trial Registry Number: CTRI/2021/11/038241.

## Introduction

Enhanced recovery after surgery (ERAS®) is a multimodal, evidence based perioperative care pathway designed to achieve early recovery for patients undergoing major surgery. The aim of implementation of ERAS protocol is to reduce the surgical stress, maintain normal physiology to expedite recovery. Enhanced recovery pathways have been developed for various orthopaedic surgeries such as hip and knee replacement [1]. lumbar spine fusion[2] as well as soft tissue sarcoma surgeries [3]. ERAS® has shown to reduce length of hospital stay and incidence of mortality and morbidity in non-oncologic orthopaedic surgeries [1, 4]. These protocols include a combination of pre-operative, intra-operative and post-operative measures.

Bone and soft tissue tumours are of various types and occur in both adults and children. These include benign, malignant and metastatic bone and soft tissue tumours [5]. Recent surgical management of musculoskeletal tumours focuses more on function preservation and quality of life [6]. Surgery often involves major blood loss and functional impairment. Adjuvant chemotherapy with its systemic complications which adds to morbidity and mortality [7]. In the context of soft tissue sarcomas, studies have demonstrated that ERAS® protocols are associated with lower morbidity and shorter hospital stays [3]. Regarding bone tumours, evidence is more limited. However, some studies suggest that adherence to ERAS® protocols can decrease length of stay, postoperative nausea and vomiting, postoperative pain, blood transfusion requirements, and 24-h drainage in bone tumour surgeries [1, 4, 8].

Despite its proven role in soft tissue sarcomas and spinal tumours, the application of ERAS® protocols in bone tumour surgeries remains understudied. This study hypothesises that ERAS® implementation in surgeries for bone and soft tissue tumours can reduce postoperative complications and shorten hospital stays.

## Methods

This prospective observational cohort study was conducted after approval from the Institutional Ethics Committee and registration with the Clinical Trial Registry of India (CTRI/2021/11/038241). All consecutive adult patients aged more than 18 years, American Society of Anesthesiologists class I-III, planned for bone and soft tissue tumour (benign or malignant) of upper limb, lower limb, spine and pelvis (with or without neo-adjuvant chemotherapy) were recruited after a written informed consent. Exclusion criteria were patients posted for emergency surgeries and intraoperative decision of inoperability. Preoperative assessment and optimization of all the eligible patients was done by concerned team. Intraoperative and post-operative management and analgesia were done as per standard of care and institutional protocols. Compliance of each patient to the following 20 proposed ERAS® components for bone and soft tissues onco-surgeries was measured preoperatively, intra-operatively and postoperatively (Table 1). All the authors of this study are good clinical practice trained and the study is conducted as per the guidelines of Declaration of Helsinki 2013 and its later amendments.

**Table 1:** Elements of ERAS® Protocol evaluates and definitions of compliance:

Sr. No	ERAS® component	Compliance
1	Counselling-Alcohol	Advised to quit in preoperative visit
2	Counselling- Smoking	Advised to quit in preoperative visit
3	Physiotherapy consultation	Consulted 7 days prior to surgery
4	Correction of anaemia	Corrective measures of anaemia in the form of oral/parenteral haematinics
5	Albumin correction	Serum Albumin > 3.5 g/dl or Corrective measures in the form of high protein diet/dietician consultation/albumin transfusion in patients with serum Albumin < 3.5 g/dl
6	Fasting- solids	Six hours before the surgery
7	Fasting- Liquids	Clear liquid 2 h before surgery
8	Carbohydrate loading	Two hours prior to the surgery
9	Pre-operative antibiotic prophylaxis	Intravenous antibiotics before skin incision
10	Chlorhexidine scrub bath	Taken on the day of surgery
11	Anti-microbial skin preparation	With chlorhexidine containing solutions preoperatively
12	Intraoperative normothermia	Maintained by active warming (warming blankets/Warm IV fluids)
13	Early mobilization	Within 24 h of the surgery
14	Early initiation of physiotherapy	Initiated on the day of surgery
15	Multimodal analgesia	≥ 2 different analgesics or modes of analgesia
16	Limited surgical drains	< 2 drains
17	Early removal of surgical drains	Removed < 72 h of surgery
18	Early catheter removal	Not catheterized/removed < 24 h of surgery
19	Resumption of oral liquid	Resumed < 12 Hours of surgery
20	Resumption of normal diet	Resumed < 24 h of surgery

The primary objective of this study was to assess the percentage compliance with individual components of the bone and soft tissue surgery enhanced recovery pathway protocols. The secondary objectives were to assess the average compliance of patients with the components of the bone and soft tissue enhanced recovery pathway and to compare postoperative complications among patients with varying levels of compliance with the bone and soft tissue enhanced recovery pathway.

Intraoperative and postoperative complications, including bleeding, re-surgery, surgical site infections, wound dehiscence, ileus, and hospital length of stay were documented. Complications were classified according to the Clavien-Dindo class of complications [9], additionally non-surgical complications such as cardiovascular, respiratory, urinary, gastrointestinal issues, and postoperative venous thromboembolism. All the patients were monitored postoperatively until discharge or death in hospital. A 30-day follow-up was conducted to assess hospital readmissions and mortality.

**Statistical analysis:** Descriptive statistics were used to summarize baseline characteristics, with continuous variables presented as mean ± standard deviation (SD) and categorical variables as frequencies and percentages. The normality of data was assessed using the Shapiro–wilk test. For inferential analysis, Pearson correlation was used to assess the linear relationship between ERAS® compliance percentage and postoperative complications (Clavien- dindo grading), hospital stay, readmissions, the receiver operating curve (ROC) analysis was done to identify the cut off for compliance to ERAS® factors affecting post operative complications graded as Clavien dindo Classification. Chi square testing was done to assess the association between overall compliance percentage (high Vs Low) and postoperative complications (Clavien-dindo class), along with compliance in different type of tumour and postoperative complications, Hospital stays, readmissions. Logistic regression analysis was done to identify significant factors associated with post operative complications.

## Results

A total of 300 patients from December 2021 to May 2023 were included in the study, comprising 180 males (60%) and 120 females (40%). The median age was 34 years (range: 18–80 years). The median body mass index (BMI) was 22.4 kg/m^2^ (range: 12.3–35.4 kg/m^2^) (Table 2).

**Table 2:** Demographic, Tumour, and Surgical Characteristics

Characteristic	Value
**Age (years)**	34 (range: 18–80)
**Sex**	
- Male	180 (60%)
- Female	120 (40%)
**Body Mass Index (kg/m**^**2**^**)**	22.4 (range: 12.3–35.4)
**ASA Status**	
- ASA 1	164 (54.7%)
- ASA 2	131 (43.7%)
- ASA 3	5 (1.7%)
**Tumour Location**	
- Lower limb	215 (71.7%)
- Upper limb	44 (14.7%)
- Pelvis	29 (9.7%)
- Back and spine	9 (3%)
- Others	3 (1%)
**Preoperative Treatment**	
- Chemotherapy	141 (47%)
- Radiotherapy	0 (0%)
**Tumour Type**	
- Primary bone tumours	160 (53.3%)
- Soft tissue tumours	140 (46.7%)
**Histological Subtypes**	
- Osteogenic Sarcoma	76 (25.3%)
- Giant Cell Tumour	54 (18%)
- Chondrosarcoma	29 (9.7%)
- Ewing’s Sarcoma	14 (4.7%)
- Spindle Cell Sarcoma	20 (6.7%)
- Synovial Sarcoma	15 (5%)
- Squamous Cell Sarcoma	12 (4%)
- Pleomorphic Sarcoma	15 (5%)
- Rhabdomyosarcoma	5 (1.7%)
- Others	60 (20%)
**Surgical Procedures**	
- Wide Local Excision with Primary Closure	89 (29.7%)
- Wide Local Excision with Arthroplasty	66 (22%)
- Amputation Surgery	61 (20.3%)
- Wide Local Excision with Plastic Reconstruction	38 (12.7%)
- Others	46 (15.3%)

Of the 300 patients, 215 (71.7%) had tumours in the lower limb, 44 (14.7%) in the upper limb, 29 (9.7%) in the pelvis, nine (3%) in the back and spine, and three (1%) in the head and neck soft tissue tumours (supraclavicular, scapular and clavicle area).

Regarding tumour types, 160 patients (53.3%) had primary bone tumours, and 140 (46.7%) had soft tissue tumours. The most prevalent histological subtypes were osteogenic sarcoma (76 patients, 25.3%) and giant cell tumours (54 patients, 18%) (Table 2).

The surgical procedures performed included wide local excision (WLE) with primary closure in 89 patients (29.7%), WLE with arthroplasty in 66 (22%), amputation in 61 (20.3%), WLE with plastic reconstruction in 38 (12.7%), and other in 46 (15.3%) patients (Table 2).

Overall compliance rate was 81.8% and 82.2% in in bone tumours and soft tissue tumours respectively. Univariate analysis using the Chi-square test showed a statistically significant association between tumour type and postoperative complications (χ^2^ = 3.984, p = 0.04), with soft tissue tumours associated with a higher risk of complications. Notably, in multivariate analysis, tumour type (bone vs soft tissue) was not an independent predictor of major postoperative complications.

Compliance with individual ERAS® protocol components is summarised in (Table 3). Preoperatively, 100% compliance was achieved for counselling on alcohol and smoking cessation. Compliance with preoperative physiotherapy consultation was 99.3%. Among the 300 patients, 71 (23.5%) were found to be anaemic. Haematinics were administered to 36 of these 71 patients, resulting in a compliance rate of 50.7% for anaemia correction. Nutritional optimisation achieved a compliance rate of 90.7%. Additionally, 54 patients (17.9%) had serum albumin < 3.5 g/dl, of whom 25 received nutritional intervention.

**Table 3:** Compliance with Enhanced Recovery After Surgery (ERAS®) components

ERAS® Component	Compliance Percentage
1) Alcohol Abstinence	100
2) Smoking Abstinence	100
3) Physiotherapy consultation	99.3
4) Anaemia Correction	50.7
5) Albumin Correction	90.7
6) Fasting for solids for 6 h	99
7) Fasting for Liquids for 2 h	82.7
8) Preoperative Carbohydrate loading	77.3
9) Chlorhexidine bath	100
10) Antimicrobial Skin Preparation	100
11) Preoperative Antibiotic prophylaxis	100
12) Maintenance of Normothermia	100
13) Early mobilisation (< 24 h of the surgery)	70.3
14) Active Limb Physiotherapy	70.3
15) Post operative multimodal analgesia	98.7
16) Limited surgical drains (< 2 drains)	70.7
17) Early drain removal (< 72 h)	33.3
18) Early catheter removal (< 24 h)	81
19) Early resumption of Oral fluids (< 12 h)	89.7
20) Early resumption of Normal diet (< 24 h)	76.3

*ERAS*®: Enhanced recovery after surgery

Perioperative compliance to enhanced recovery pathway components were as follows: fasting for liquids for 2 h (82.7%) and solids for 6 h (99%), preoperative carbohydrate loading (77.3%), chlorhexidine bathing (100%), antimicrobial skin preparation (100%), antibiotic prophylaxis (100%), and maintenance of intraoperative normothermia (100%).

Postoperatively, 211 patients (70.3%) were mobilised with limb physiotherapy within 24 h of the surgery. Multimodal analgesia for postoperative pain was administered to 296 patients (98.7%). The proportion of patients with fewer than two surgical drains was 70.7%, but compliance with early drain removal (< 72 h) was 33.3%. Early urinary catheter removal (< 24 h) achieved 80% compliance.

Surgical complications were classified using the Clavien-Dindo class. Class 1 and 2 were marked as minor complications, class 3 and 4 as major complications, and class 5 referred to in-hospital mortality (Table 4).

**Table 4:** Postoperative Outcomes, Correlation, and Multivariate Analysis

Variable		Value/Correlation (r)/Coefficient	p-value
**Clavien-Dindo Class**		**n (%)**	
No Complications (Class 0)		38 (12.6%)	—
- Class I-II		245 (81.7%)	—
- Class III-IV		16 (5.3%)	—
- Class V		1 (0.3%)	—
**Pearson Correlation with Compliance**	**Type of tumour**	—	—
-Postoperative Complications (Clavien-Dindo)	Bone tumours	**r = −0.235**	**0.003**
	Soft tissue tumours	**r = −0.393**	**0.000**
- Hospital Stay	Bone tumours	**r = −0.426**	**0.000**
	Soft tissue tumours	**r = −0.170**	**0.044**
- Readmissions	Bone tumours	**r = −0.179**	**0.024**
	Soft tissue tumours	**r = −0.155**	**0.067**
**Multivariate Analysis**		**Coefficient**	**p-value**
- Age		**0.214**	**0.001**
- Body Mass Index		−0.018	0.741
- ASA		0.057	0.313
- Site of Tumour		0.064	0.232
- Tumour Type		−0.027	0.661
- Preoperative Hemoglobin		0.022	0.722
- Preoperative Albumin		−0.006	0.928
- Duration of Surgery		**0.189**	**0.004**
- Blood Loss		0.103	0.123
- Compliance to ERAS® Component		**−0.219**	**0.000**

*ASA*: American Society of Anesthesiologist, *BMI*: Body Mass Index

There was a significant association between tumour type and the occurrence of major postoperative complications (Clavien-Dindo grade ≥ III), with patients diagnosed with soft tissue tumours demonstrating a higher risk of complications compared to those with bone tumours (Table 5).

**Table 5:** Association Between Tumour Type and Severity of Postoperative Complications

Test	Chi-Square Value	df	P Value (2-sided)
Pearson Chi-Square	3.984	1	0.046 *

Statistically significant at *p *< 0.05

Statistically significant associations were observed for most outcomes in both tumour groups, with the strongest correlation between compliance and reduced hospital stay in patients with bone tumours (r = –0.426, p < 0.001). The hospital readmission correlation in soft tissue tumours was not statistically significant (p = 0.067).(Table 4).

Median hospital stay in our study was 8 days with the range of (1–68 days). We found a statistically significant difference and negative correlation between the compliance to the ERAS® components and length of hospital stay (p < 0.000) with a Pearson correlation coefficient of −0.325 (Table 4).

Nineteen patients were readmitted to hospital within 30 days of surgery, and we found a statistically significant difference and negative correlation between the compliance to the ERAS® components and readmission to the hospital (p = 0.026%) with a Pearson correlation coefficient of −0.128 (Table 4).

Multivariate logistic regression analysis was conducted to evaluate the association between improved compliance with ERAS® components and reduced postoperative morbidity. Factors considered included age, BMI, preoperative haemoglobin, preoperative albumin, duration of surgery, blood loss, tumour type, tumour site, and ERAS® compliance percentage (Table 4).

**Fig. 1:**
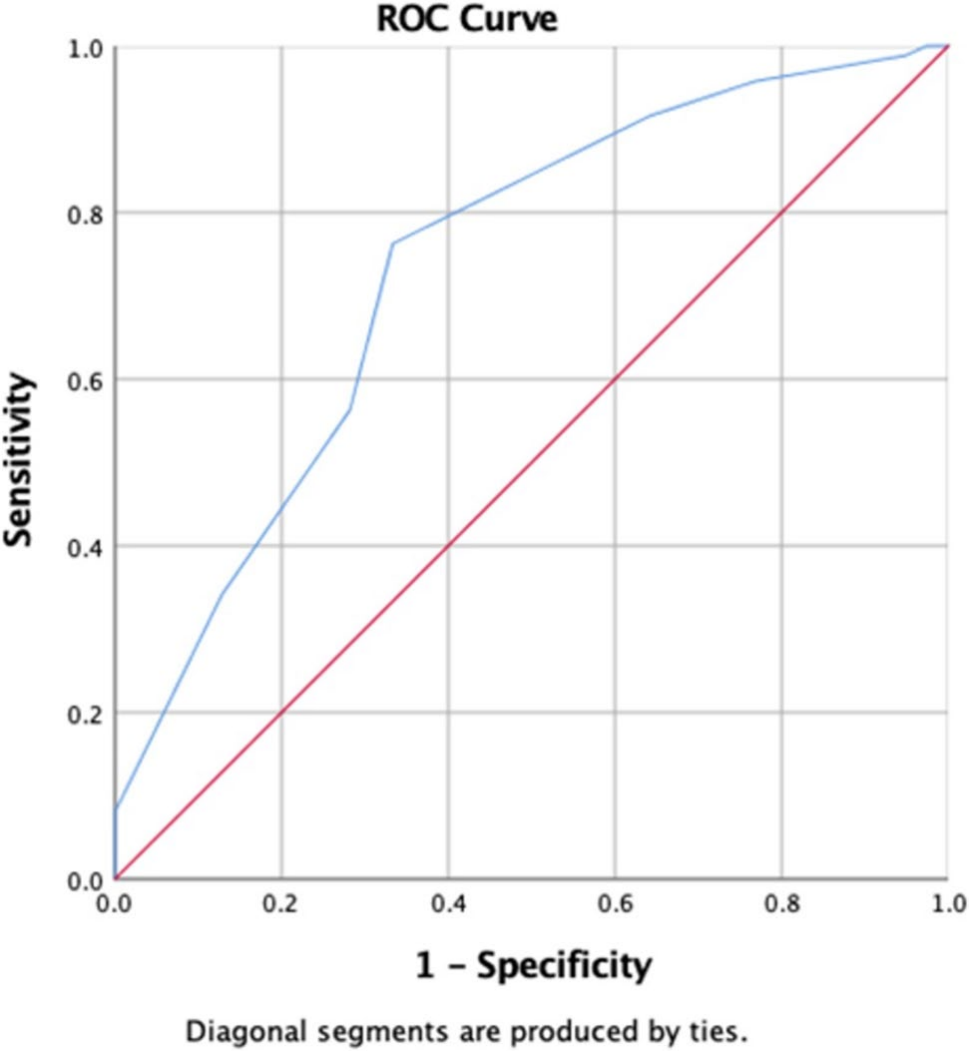
The receiver operating curve of compliance to ERAS® pathways and postoperative complications

Receiver operating characteristic (ROC) (Fig. 1) analysis identified a compliance threshold of 77.5% as the cut-off for reducing postoperative complications graded by the Clavien-Dindo classification. The area under the curve (AUC) was 0.735 (p < 0.000), indicating good discriminatory power of ERAS® compliance in predicting postoperative outcomes.

## Discussion

The ERAS® protocols were developed for the purpose of hastening functional recovery after surgery, reducing the morbidity associated with surgery and minimizing the length of hospital stay. This is achieved by reducing the stress associated with surgery and by returning the patient to their normal physiology as soon as possible following surgery. Previous studies have shown ERAS® protocols to be successful in achieving this aim [10–12].

Our research identified differential compliance with specific elements of the bone and soft tissue enhanced recovery pathway. The highly compliant elements (compliance > 77.5%) were preoperative lifestyle changes, correction of hypoalbuminemia, physical exercises, chlorhexidine bathing, antimicrobial skin preparation, and antibiotic prophylaxis. Compliance was comparatively lower for correction of anemia, early mobilization after surgery, limb physiotherapy, restricted drain use, early catheter removal, early drain removal, and early return to a normal diet.

Low adherence to the correction of anemia can be explained by the time-critical nature of these operations and the nutritional deficiencies from preoperative chemotherapy that limit the window for effective intervention. The complexity of cases, with extensive tissue manipulation and reconstruction in amputations and plastic surgeries, may also contribute to low compliance with postoperative ERAS® protocols. Early mobilization and physiotherapy in certain patients with lower limb, pelvis and spine procedures were challenging in view of gait instability from extensive resections, significant postoperative pain (particularly after pelvic or spinal surgeries), reconstructive procedures requiring immobilization, presence of multiple drains or external fixators, and muscle weakness due to tumour involvement, nerve injury, or excision of major muscle groups along with preoperative poor nutritional status, preoperative chemotherapy. Despite these challenges, early mobilization was prioritized. Our institution has a dedicated acute pain service and a multidisciplinary rehabilitation team including physiotherapists, who initiated mobilization as early as feasible and provided continuous postoperative support for physiotherapy. Notably, patients with higher ERAS® compliance (≥ 77.5%) exhibited fewer postoperative complications (AUC = 0.7, p < 0.0001).

Previous studies in orthopaedic surgery have demonstrated that high compliance with enhanced recovery protocols is associated with early functional recovery and early discharge after surgery. [4] Other studies involving oncologic surgeries have also reported lower major complication rates and a decreased length of hospital stay [10, 11]..

We compared the post-operative complications between patients with different levels of compliance to the bone and soft tissue enhanced recovery pathway. Out of 300 patients, 245(81.7%) patients experienced minor postoperative complications (Clavien Dindo I-II) while 17 (5.6%) patients experienced major complications (Clavien Dindo III-V). There was a statistically significant negative correlation (p < 0.000) between both the total and percentage compliance to the 20 ERAS® pathway components and the severity of postoperative complications. The Pearson correlation coefficient was −0.235 for bone tumours and −0.393 for soft tissue tumours. We also found a higher incidence of major postoperative complications (Clavien-Dindo grade ≥ III), with soft tissue tumours being associated with a significantly increased risk (p < 0.046). Similar results were obtained in study of ERAS® in soft tissue sarcoma; wound dehiscence rate was lower in ERAS group (0⋅9%) vs (13.1%) with (P < 0.001) in control group [3].

A compliance study of ERAS® protocol in colorectal cancer surgeries found the similar results, they stated that patients with highest compliance has surgical site wound infection rate as 6.9% (p < 0.05%), which was significant as compared to other groups of various degree of compliance [10].

Our study found that the overall compliance rate with ERAS® protocols among patients undergoing bone and soft tissue cancer surgeries was 84.5%. Specifically, the compliance rate was 81.8% in the bone tumour group and 82.2% in the soft tissue tumour group. Although few studies have specifically examined ERAS® in bone and soft tissue cancer surgeries, our findings are consistent with research on ERAS® in other orthopaedic surgeries [4] and other cancer surgeries [11], where higher compliance has been associated with fewer major postoperative complications and shorter hospital stays. To our knowledge, this is the first study to directly assess ERAS® compliance in this specific patient population.

A prospective study, was done to evaluate safety and efficacy of ERAS® in intraspinal tumours, with primary outcome as length of hospital stay (LOS) [12], They found that median LOS was significantly low in the ERAS® group (5 days) as compared to the group following standard institutional perioperative protocol group (8 days). Similar results were found by Lyu et al.[3] in their implementation of ERAS® in patients with soft tissue sarcoma.

Median hospital stay in our study was 8 days with the range of (1-68 days). We found a significant difference with negative correlation between the compliance to the ERAS® components and Length of hospital stay with the strongest correlation between compliance and reduced hospital stay in patients with bone tumours (r = –0.426, p < 0.000)..

Nineteen patients were readmitted to hospital within 30 days of surgery, and we found a significant difference and negative correlation between the compliance to the ERAS components and readmission to the hospital (p < 0.024%) with a Pearson correlation of −0.179in bone tumours. Readmission correlation in soft tissue tumours was not statistically significant (p = 0.067). In a preliminary analysis of implementation of ERAS® protocol in spine metastatic surgery in a cancer centre has shown a higher rate of 30-day readmission in Pre-ERAS® group as compared to ERAS® group [13].

Although our study yields useful information on compliance and effectiveness of ERAS® protocols for bone and soft tissue cancer resections, several limitations need to be recognised. Firstly, it is a single-centre study, which could make generalisation of our results to other institutions with various patient groups and perioperative regimens limited. Second, compliance rates were self-reported by the healthcare teams, with the resultant risk of reporting bias. Third, although we noted substantial correlations between ERAS® compliance and better outcomes, causality cannot be conclusively ascertained because the study is observational. Finally, heterogeneity of patients by tumour type, surgical complexity, and preoperative health status could have affected the results and required further stratified analyses.

## Conclusion

Higher ERAS® compliance was associated with shorter hospital stays and fewer complications, suggesting its potential of better postoperative outcomes in bone and soft tissue cancer surgeries. Further studies stratifying patients by surgicalcomplexity are needed to confirm these findings.
